# Regulated secretion of mutant p53 negatively affects T lymphocytes in the tumor microenvironment

**DOI:** 10.1038/s41388-023-02886-1

**Published:** 2023-11-11

**Authors:** Xiang Dong, Chunlu Li, Chengsi Deng, Jingwei Liu, Danni Li, Tingting Zhou, Xindi Yang, Yunchan Liu, Qiqiang Guo, Yanling Feng, Yang Yu, Zhuo Wang, Wendong Guo, Siyi Zhang, Hongyan Cui, Cui Jiang, Xiwen Wang, Xiaoyu Song, Xun Sun, Liu Cao

**Affiliations:** 1 The College of Basic Medical Science, Health Sciences Institute, China Medical University, Shenyang, Liaoning Province China; 2 Key Laboratory of Medical Cell Biology, Key Laboratory of Precision Diagnosis and Treatment of Gastrointestinal Tumors, Ministry of Education, China Medical University, Shenyang, China; 3 Department of Medical Oncology, the First Affiliated Hospital of China Medical University, Shenyang, Liaoning Province China; 4 Department of Medical Oncology, Cancer Hospital of China Medical University, Liaoning Cancer Hospital and & Institute, Shenyang, Liaoning Province China; 5 Department of Thoracic Surgery, Shengjing Hospital of China Medical University, Shenyang, Liaoning China

**Keywords:** Protein transport, Oncogenes

## Abstract

Several studies have demonstrated the role of the oncogenic mutant p53 in promoting tumor progression; however, there is limited information on the effects of secreted oncogenic mutant p53 on the tumor microenvironment and tumor immune escape. In this study, we found that secretion of mutant p53, determined by exosome content, is dependent on its N-terminal dileucine motif via its binding to β-adaptin, and inhibited by the CHK2-mediated-Ser 20 phosphorylation. Moreover, we observed that the mutant p53 caused downregulation and dysfunction of CD4^+^ T lymphocytes in vivo and downregulated the levels and activities of rate-limiting glycolytic enzymes in vitro. Furthermore, inhibition of mutant p53 secretion by knocking down AP1B1 or mutation of dileucine motif could reverse the quantity and function of CD4^+^ T lymphocytes and restrain the tumor growth. Our study demonstrates that the tumor-derived exosome-mediated secretion of oncogenic mutant p53 inhibits glycolysis to alter the immune microenvironment via functional suppression of CD4^+^ T cells, which may be the underlying mechanism for tumor immune escape. Therefore, targeting TDE-mediated p53 secretion may serve as a potential therapeutic target for cancer treatment.

## Introduction

Increasing evidence has demonstrated the role of tumor-derived extracellular vesicles (EVs), especially tumor-derived exosomes (TDEs), in intracellular communication during cancer progression [1, 2]. TDEs (30–100 nm) have a bilayer structure enclosing DNA, mRNA, miRNA, and oncoproteins, which are transferred to the recipient cells to remodel the tumor microenvironment (TME) by immune surveillance, pro-tumor metastasis, and chemoresistance [3–7]. Although TDEs have been studied extensively, further studies are required to explore the crosstalk between some critical TDE contents and TME remodeling.

p53, a well-known tumor suppressor and transcription factor, regulates multiple biological processes, including the cell cycle, senescence, and apoptosis [8–10]. However, *TP53* gene mutations have been identified in >50% of human cancers, including colorectal cancer, pancreatic cancer, and lung cancer [11, 12]. The oncogenic gain of function p53 mutant, known as mutant p53, shows hot spots at residues 175, 273, 248, 245, and 282 [13]. It interacts with several targets and acts as an “oncogene” via aberrant conformation or changes in DNA, causing significant alterations in the cellular pathways associated with cancer development, chemoresistance, and metabolism [14–16].

Some of the oncoproteins released into the extracellular space or TME via EVs include mutant phosphatase and tensin homolog on chromosome ten (PTEN), heat shock protein 90α (HSP90α), as well as β-catenin and its oncogenic mutant [17–21]. A study found that wildtype p53 (p53^WT^) is secreted by vesicle-like structures via the K-Ras-Snail pathway [22]. Another study demonstrated that mutant p53 secretion by EVs is dependent on HSP90α binding [23]. Although several studies have been conducted on mutant p53 secretion, its regulatory mechanism and biological functions are still being investigated.

Both WT and mutant p53 have been found to remodel the TME to regulate tumor progression. The p53^WT^ can cause cytoplasmic DNA accumulation-induced activation of the cGAS/STING signaling pathway to modulate the innate immune response, resulting in tumor suppression [24]. In contrast, the mutant p53 can promote tumor development by negatively regulating the cGAS/STING signaling pathway by hijacking the TBK1 and hindering STING-IRF3-TBK1 complex formation [25]. Although these findings have clarified the relationship between the tumor and its TME, with respect to cell autonomy and non-autonomy, the role of TDE-derived mutant p53 in TME remodeling is still not known.

In this study, we found that tumor-derived mutant p53^R273H^ is packed into the TDEs in a β-adaptin-dependent manner via its N-terminal dileucine motif. Furthermore, the CHK2-mediated phosphorylation of Ser 20 residue suppressed the secreted process. Additionally, in tumor-bearing mice, mutant p53 suppressed CD4^+^ T lymphocyte function by downregulating its metabolic changes to promote immune escape and tumor progression. More importantly, blocking the mutant p53 secretion could rescue the function of the infiltrating CD4 + T lymphocyte and alter anti-tumor microenvironment, thus leading to limiting tumor development.

## Results

### The N-terminal dileucine motif is essential for p53 sorting into the exosomes

**Fig. 1:**
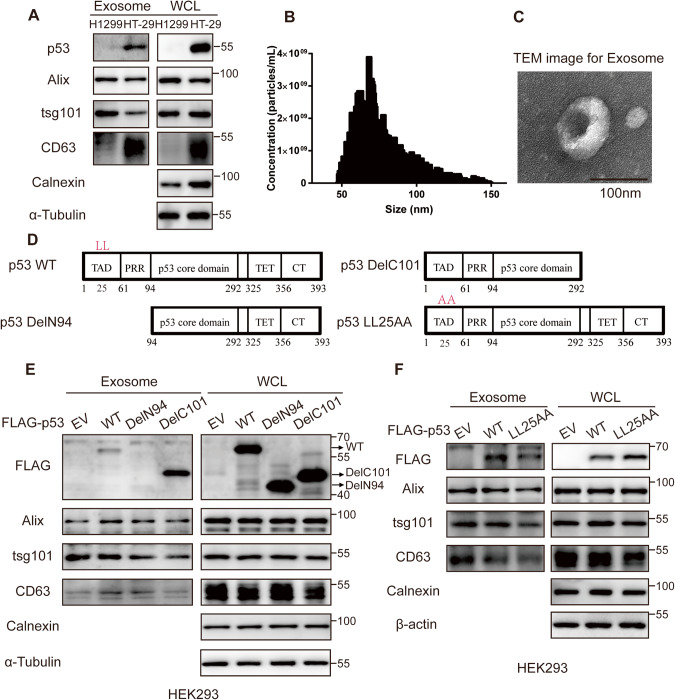
The N-terminal dileucine motif is essential for sorting p53 into the exosomes. **A** Western blot analysis of the exosomes isolated from p53^null^-expressing H1299 (Negative control) and p53^R273H^-endogenous expressing HT-29 cells, with Alix, tsg101 and CD63 considered positive markers and calnexin considered a negative marker of the exosomes. nanoparticle tracking analysis (**B**) and transmission electron microscopy analysis (**C**) of the exosomes isolated from HT-29 (p53^R273H^) cells (**C**; scale bar: 100 nm). **D** Schematic representation of the mutations in p53. **E** Expression of Flag-p53^WT^, p53^DelN94^, and p53^DelC101^ in the exosomes isolated from HEK293 cell culture supernatant. CD63, Alix, and tsg101 were considered positive markers, while calnexin was considered negative marker for exosomes. **F** Expression of Flag-p53^WT^ and p53^LL25AA^ in the exosomes isolated from HEK293 cell culture supernatant. CD63, Alix, and tsg101 were considered positive markers, while calnexin was considered negative marker for exosomes.

To verify the secretion of mutant p53, we overexpressed GFP-tagged WT and mutant p53 in H1299 cells. In addition to p53^WT^, we detected p53^R273H^, p53^R248W^_,_ and p53^R175H^ in the cell culture supernatant and α-Tubulin and β-actin in media fraction were used for negative control, indicating that mutant p53 is secreted (Fig. S1A). Thereafter, we analyzed if the secreted mutant p53 could be internalized from the extracellular media by the recipient cells. For this, HCT116 cells were cultured in conditioned media (CM), prepared by mixing fresh media with the cell culture supernatants from the cells transfected with p53^WT^ or mutant p53 plasmids. Western blot (WB) analysis of the target cell lysates revealed the presence of p53, indicating that p53 was internalized by the recipient cells (Fig. S1B). Furthermore, to determine if p53 was secreted via EVs, we conducted a WB analysis of the exosomes derived from HT-29 (p53^R273H^) and H1299 cells (p53 null) and the results revealed that p53 was only detected in the HT-29-derived EVs (Fig. 1A). Moreover, nanoparticle tracking analysis (NTA) of the HT-29-derived EVs revealed that the average size of the EVs was 77.3 nm (Fig. 1B), consistent with the size of the exosomes (30–100 nm). Additionally, transmission electron microscopy (TEM) analysis of the HT-29-derived EVs revealed a cup-shaped structure of the EVs (Fig. 1C). Altogether, these results show that p53^R273H^ is secreted from the exosomes derived from HT-29 cells.

To further investigate the underlying mechanism, we constructed two mutant forms of p53, p53^DelN94^ (lacking the N-terminal TAD and PPR domains) and p53^DelC101^ (lacking the C-terminal domain) (Fig. 1D). WB analysis revealed that the secreted form of p53^DelN94^ was significantly decreased, while p53^DelC101^ was still secreted in the exosomes or extracellular medium (Figs. 1E and S1C). These results suggest that the deletion of the N-terminal TAD and PRR domains inhibits exosome-mediated p53 secretion, while the deletion of C-terminal TET and CT domains promotes exosome-mediated p53 secretion. Furthermore, to determine if the dileucine motif in the N-terminal TAD domain is required for p53 secretion, we created a p53^LL25AA^ mutant, in which the leucine residues at 25 and 26 were changed to arginine. We found that p53^LL25AA^ showed reduced secretion in the culture medium (Figs. 1F and S1D), indicating that the N-terminal dileucine motif is required for the exosome-mediated secretion of p53.

### β-adaptin binding is required for p53 packing into the exosome

**Fig. 2:**
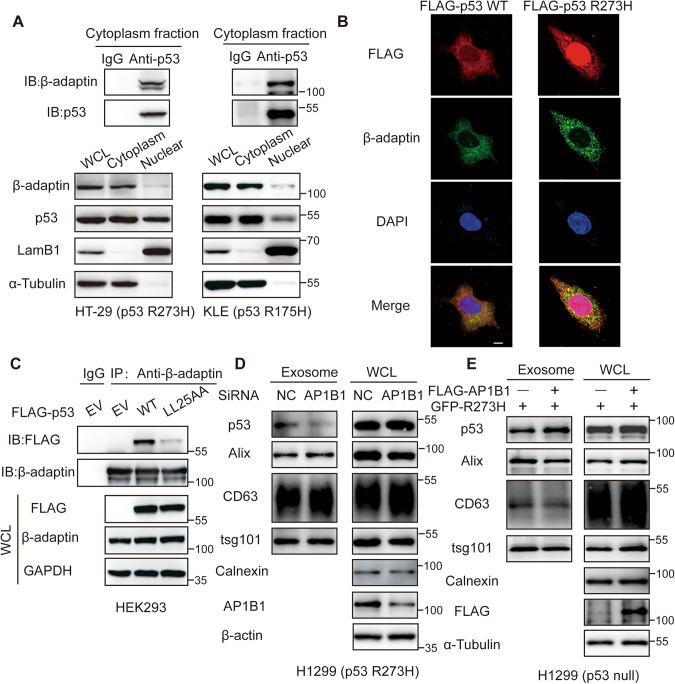
β-adaptin binding is required for p53 and mutant p53 packing into the exosomes. **A** Co-immunoprecipitation (Co-IP) analysis of the cytoplasm fraction of the endogenous p53^R273H^-expressing HT-29 and endogenous p53^R175H^-expressing KLE cells, with Lamin B1 and α-tubulin considered as the markers of nuclear and cytoplasm fractions, respectively. Anti-p53(sc-126) were used to immunoprecipitated and Anti-p53 (#2527) and anti-β-adaptin (sc-74423) were used for WB. **B** Representative images of immunofluorescence stained HEK293 cells showing endogenous β-adaptin (green), exogenous FLAG-p53^WT^ and FLG-p53^R273H^ (red), and DAPI for nuclei (blue; scale bar: 10 μm). **C** Co-IP analysis of Flag-p53^WT^ or p53^LL25AA^ mutants with endogenous β-adaptin in HEK293 cells. The whole cell lysates were extracted and immunoprecipitated using anti-β-adaptin. **D** Expression of p53^R273H^ in the exosomes isolated from the H1299 stable expressed p53^R273H^ cell culture supernatant after β1-adaptin knockdown (si-AP1B1). Alix, tsg101, and CD63 were considered as the positive markers, while calnexin was considered as a negative marker for exosomes. Anti-AP1B1 (16932-I-AP) was used to detect the silence efficiency. **E** Expression of p53^R273H^ in the exosomes isolated from the H1299 cell culture supernatant after co-transfection with exogenous GFP-p53^R273H^ and/or FLAG-AP1B1 cell culture supernatants. Alix, tsg101, and CD63 were considered as the positive markers, while calnexin was considered as a negative marker for exosomes.

The dileucine motif has been shown to regulate the intracellular trafficking of proteins along the plasma membrane–Golgi apparatus–endosome recycling pathway. For instance, the trafficking of GLUT8, STING, and EGFR is regulated by interactions between their dileucine motifs and the adaptor protein complexes associated with clathrin-coated vesicles [26–29]. Therefore, we hypothesized that p53, which also contains an N-terminal dileucine motif, serves as a “cargo” for β-adaptin. Immunoprecipitation (IP) of the endogenous p53^WT^ and p53^R273H^ in HCT116 and HT-29 cells, respectively, revealed that both the p53 proteins interacted with β-adaptin (Fig. S2A, B), and similar results were observed for the GFP-tagged mutant p53 (Fig. S2C). Additionally, we observed that β-adaptin was located in the cytosol, and co-immunoprecipitation (Co-IP) assays showed that the cytoplasmic mutants of p53, p53^R273H^ and p53^R175H^ in HT-29 and KLE cells, respectively, could bind with β-adaptin by nuclear and cytoplasm fraction separation (Fig. 2A). Moreover, confocal microscopy analysis confirmed that p53 and p53^R273H^ co-localized with β-adaptin in the cytosol (Fig. 2B). Furthermore, to assess if p53 secretion was regulated by the interaction between the dileucine motif and β-adaptin, we conducted a Co-IP analysis of p53^LL25AA^ and p53^WT^ and found that, compared with p53^WT^, p53^LL25AA^ decreased obviously the ability to interact with β-adaptin (Fig. 2C). Subsequently, the silencing of β1-adaptin, by small interfering RNA, led to a decrease in the packaging of p53^R273H^ into the exosomes, compared with the control (Fig. 2D). Conversely, the level of exosome-derived p53^R273H^ was increased in the β1-adaptin-overexpressing cells, compared with the control cells (Fig. 2E). These results demonstrate that the N-terminal dileucine motif in p53 is required for its interaction with β-adaptin and its sorting into the exosomes.

### CHK2-mediated phosphorylation of mutant p53 Ser20 inhibits secretion

**Fig. 3:**
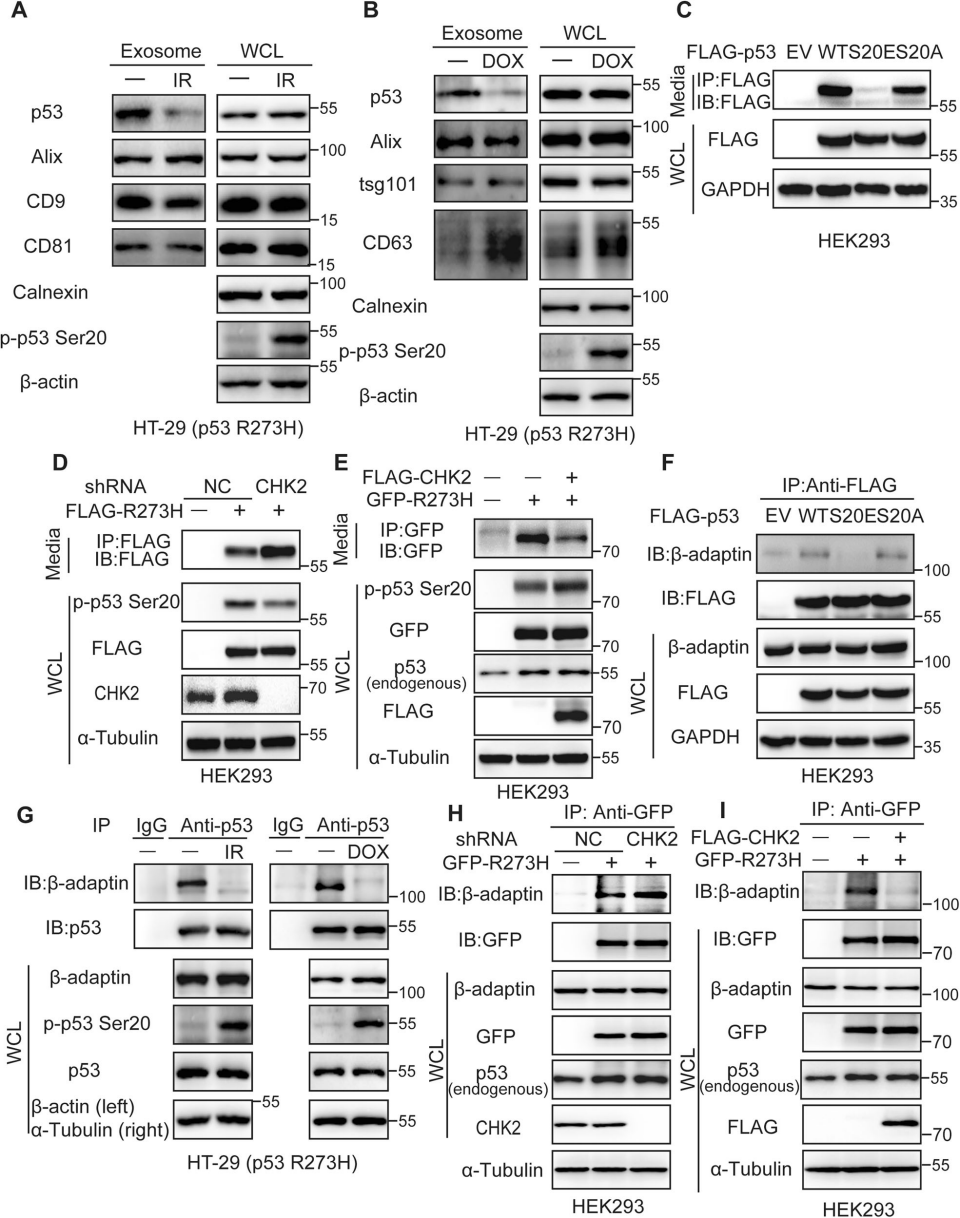
CHK2-mediated phosphorylation of mutant p53 Ser20 inhibits secretion. Expression of endogenous p53^R273H^ in the exosomes isolated from the HT-29 (p53^R273H^) cell culture supernatant after treatment without or with post-10Gy IR for 4 h (**A**) and 500 nM DOX for 12 h (**B**). Alix, tsg101, CD9, CD81 and CD63 were considered as the positive markers, while calnexin was considered as a negative marker for exosomes. **C** Expression of exogenous p53 mutations immunoprecipitated by anti-FLAG from the HEK293 cell culture supernatant after transfection with FLAG-p53^WT^, FLAG-p53^S20E^ and FLAG-p53^S20A^. Expression of exogenous p53^R273H^ immunoprecipitated by anti-FLAG or anti-GFP from the HEK293 cell culture supernatant after transfection with FLAG-p53^R273H^/GFP-p53^R273H^ cell culture supernatants after knocking-down CHK2 (**D**) or overexpression of FLAG-CHK2 (**E**). **F** Co-IP analysis of FLAG-p53^WT^, FLAG-p53^S20E^ and FLAG-p53^S20A^ mutants with endogenous β-adaptin in HEK293 cells. The whole cell lysates were extracted and immunoprecipitated using anti-FLAG. **G** Expression of endogenous p53 and β-adaptin using anti-p53 to perform Co-IP assay in HT-29 (p53^R273H^) cell after treatment without or with 10 Gy IR (left) and 500 nM DOX (right). Expression of p53 and β-adaptin using anti-GFP to perform Co-IP assay in HEK293 cells after transfection with GFP-p53^R273H^ and knocking-down of CHK2 (**H**) or overexpression of FLAG-CHK2 (**I**).

Phosphorylation of p53 is essential for its activity and function and is therefore one of the most important posttranslational modifications on the p53 protein [30]. We determined if the phosphorylation status of Ser20, which close to the dileucine motif, played any role in regulating mutant p53 secretion. Firstly, we treated with DOX and ionizing radiation (IR) in HT-29 (p53^R273H^) cells. The stress conditions caused upregulation of p-p53 Ser20, but strongly decreased the secretion behavior of mutant p53 via exosome (Fig. 3A, B). Similar results were obtained in the media fraction of H1299 stable overexpression of p53^R273H^ cells (Fig. S3A, B). Subsequently, we introduced the S20A mutation to mimic loss of phosphorylation activity, or the S20E to mimic gain of phosphorylation activity. Whereas WT p53 containing S20E was remarkably decreased the secretion behavior, WT p53 bearing S20A was secreted similarly to WT p53 (Fig. 3C). These results indicate that the phosphorylation at residue Ser20 is a key mechanism that regulates mutant p53 secretion.

We next asked if CHK2, which catalyzes the phosphorylation of p53 WT at residue Ser20 [31], is involved in mutant p53 secretion. We first confirmed that p53^R273H^ is phosphorylated by CHK2 under stress conditions, such as DOX treatment, similarly to WT p53 (Fig. S3C). Silencing CHK2 expression by shRNA corresponded with an increase in the secretion of mutant p53 compared with the control (Fig. 3D). To rule out the possibility of shCHK2-offtarget effects, we detected that re-overexpression of CHK2 in 293 shCHK2 cells reversed the secretion behavior of p53^R273H^ (Fig. S3D). On the other hand, overexpression of CHK2 led to a significant decrease in the level of mutant p53 in the extracellular media compared with the control (Fig. 3E). Taken together, these observations indicate that CHK2, which regulates p53 Ser20 phosphorylation, inhibits the secretion of mutant p53.

We then examined the link between the N-terminal dileucine motif/β-adaptin interaction and Ser20 phosphorylation dependent p53^R273H^ secretion. To address the point, cells were transfected with the S20E or S20A mutants and the results were that the phosphomimic mutant S20E clearly showed no interaction with β-adaptin, whereas the non-phosphomimic mutant S20A and WT showed similar ability to interact with β-adaptin (Fig. 3F). Similarly, treatment with IR and doxorubicin increased phosphorylation of p53^R273H^ Ser20 and, interestingly, decreased the interaction between p53 and β-adaptin relative to the control (Fig. 3G). Moreover, knockdown of CHK2 resulted in an increase in p53^R273H^ binding to β-adaptin (Fig. 3H). Conversely, overexpression of CHK2 disrupted the interaction between p53^R273H^ and β-adaptin (Fig. 3I). Collectively, these results provide strong evidence that CHK2 mediated-phosphorylation of mutant p53 Ser20 negatively regulates its secretion by inhibiting interaction with β-adaptin.

### Mutant p53 promotes the immunosuppressive status of CD4^+^ T lymphocytes in the TME

**Fig. 4:**
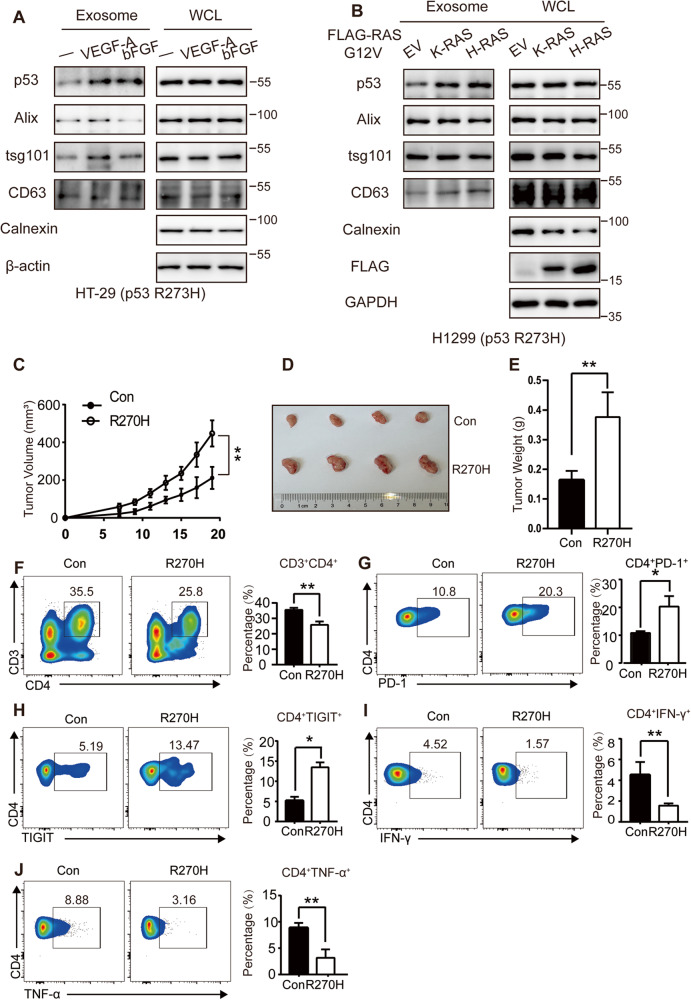
Mutant p53 promotes the immunosuppressive status of CD4^+^ T lymphocytes in the tumor microenvironment. **A** Expression of endogenous p53^R273H^ in the exosomes isolated from HT-29 cell culture supernatant after pretreatment with serum starvation for 4 h and subsequent treatment with 10 ng/ml VEGF-A or 50 ng/ml bFGF for 12 h. **B** Expression of p53^R273H^ in the exosomes isolated from H1299 stable expressed p53^R273H^ cell culture supernatant after transfection with control, FLAG-K-RAS G12V, or H-RAS G12V plasmids. **C** Graphical quantification represents tumor growth rate in mice with time (*n* = 4) after an equal amount of 4T-1 control, Trp53^R270H^-expressing cells were injected into the mammary gland of female BALB/c mice. **D** 4T-1 tumor-harboring mice were sacrificed on day 19. Representative images show tumor volume difference between Con and R270H. **E** Graphical quantification of the difference in tumor weight between Control and R270H mice (*n* = 4). **F** Flow cytometry analysis of CD45, CD3, and CD4 in T lymphocytes and the percentage of CD3^+^ CD4^+^ T lymphocytes isolated from R270H and its control mice. **G** Flow cytometry analysis of CD45, CD4, and PD-1 in T lymphocytes and the percentage of CD4^+^ PD1^+^ T lymphocytes isolated from R270H and its control mice. **H** Flow cytometry analysis of CD45, CD4, and TIGIT in T lymphocytes and the percentage of CD4^+^ TIGIT1^+^ T lymphocytes isolated from R270H and its control mice. Quantification of Interferon-γ^+^ (**I**) and tumor necrosis factor-α^+^ (**J**) cells as a percentage of CD4^+^ T cells, after 4 h of stimulation with PMA/Ionomycin, isolated from R270H and control mice.

We further explored if p53 secretion is regulated in TME and observed that TDE-derived-secretion of p53^R273H^ increased after treatment with extracellular cytokines, including VEGF-A and bFGF (Fig. 4A) and transfection with K-Ras G12V or H-Ras G12V, which are associated with oncogenic mutation induced-activation (Fig. 4B). Similar results were also observed in the same cell culture supernatant by immunoprecipitated with anti-p53 antibodies (Fig. S4A, B). These results indicate that the secretion of mutant p53 is dynamically regulated in the TME. Furthermore, to determine the role of mutant p53 in the TME, we engineered 4T-1 cells (murine breast cancer cells) to overexpress trp53^R270H^ (p53^R273H^ in human), trp53^R172H^ (p53^R175H^ in human) and their respective negative control group detected by WB (Fig. S4C) and injected an equal amount of negative control and trp53^R270H^/trp53^R172H^ -expressing 4T-1 cells into the mammary fat pad of BALB/c mice. After 19 d of transplantation, both the tumor in the p53^R270H^ and p53^R172H^-injected mice (R270H or R172H mice) showed a faster growth rate compared with the negative control -injected mice (Figs. 4C and S4E). Moreover, the weight of the R270H or R172H mice was much heavier than their Control mice (Figs. 4E and S4H) and their representative images of tumors and their H&E staining in the above groups were shown in Figs. 4D, S4D, F and G.

Subsequently, we conducted a flow cytometry analysis to determine if the tumor-infiltrating CD4^+^ T cells were affected by mutant p53 secretion and the results revealed that the frequency of CD3^+^ CD4^+^ T cells was decreased in the R270H or R172H mice, compared with the control (Figs. 4F and S4I). The surface expression of PD-1 and TIGIT was used as a marker of T cell exhaustion, and the frequency of CD4^+^ PD-1^+^ and CD4^+^TIGIT^+^ cells was markedly increased in both R270H and R172H mice, compared with the Control mice (Figs. 4G, H, S4J, K), suggesting that the CD4^+^ T lymphocytes in mutant p53 mice lost their anti-tumor activity. Furthermore, intracellular interferon-γ (IFN-γ) and tumor necrosis factor-α (TNF-α) levels were decreased in the R270H and R172H mice compared with the Control mice by flow cytometry analysis (Figs. 4I, J, S4L, M). Altogether these results demonstrate that the tumor-infiltrating CD4^+^ T cells in mutant p53 mice showed low-activity and pro-tumor characteristics, suggesting that the mutant p53 significantly exhausts the immunosuppressive status of CD4^+^ T cells, thus causing tumor immune evasion.

### Tumor-derived mutant p53 inhibits glycolysis and promotes apoptosis under metabolic stress in T lymphocytes

**Fig. 5:**
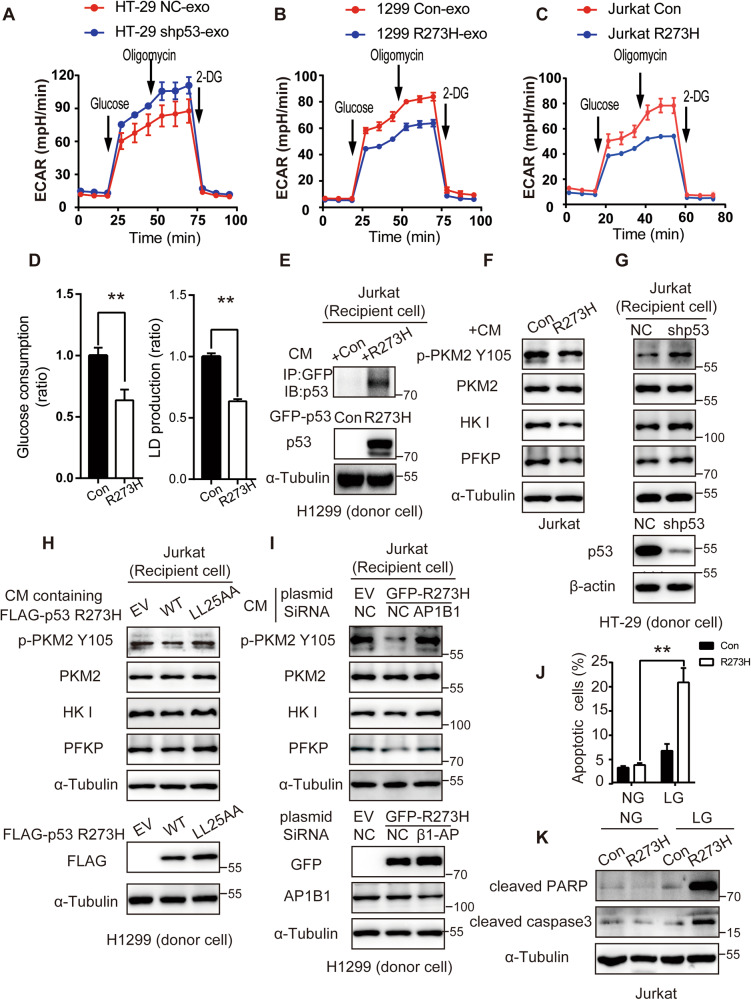
Tumor-derived mutant p53 inhibits glycolysis and promotes apoptosis under metabolic stress in Jurkat T lymphocytes. **A** Dynamic monitoring of extracellular acidification rate (ECAR), as the parameters of glycolytic flux, were measured by the XF Glycolysis Stress Test Kit, after the Jurkat cells pretreatment with HT-29 NC or HT-29 shp53 cells derived exosomes for 18 h (4 μg/ml). **B** Seahorse tracing curves for ECAR in Jurkat cells that were treated with exosomes (4 μg/ml) from Con or p53^R273H^ OE H1299 cells for 18 h. **C** Seahorse tracing curves for ECAR in Con or p53^R273H^ OE Jurkat cells. **D** Analysis of glucose consumption and lactate production in Jurkat Con or R273H cells according to the instructions. **E** A co-culture system was used to detect the uptake of H1299-secreted GFP-p53^R273H^ (lower layer) into the Jurkat cells (upper layer) over 24 h of incubation. The internalization of GFP-p53 was detected by immunoprecipitation of recipient cell lysates using anti-GFP antibodies, followed by WB analysis. **F** WB analysis of glycolysis markers (p-PKM2 (Y105), PKM2, HK I, PFKP) in the recipient Jurkat cells after co-cultured with Con or p53^R273H^ OE H1299 cells by co-culture chamber system for 24 h. **G** WB analysis of glycolysis change (p-PKM2 (Y105), PKM2, HK-I, PFKP) in the recipient Jurkat cells after co-cultured with HT-29 NC or HT-29 shp53 cells by co-culture chamber system for 24 h. **H** The glycolysis change of Jurkat cells, detected by the p-PKM2 (Y105), PKM2, HK I, PFKP, after co-cultured with H1299 empty vector (EV), Flag-p53^R273H^ or p53^R273H LL25AA^ for 24 h. **I** The glycolysis markers of Jurkat cells, detected by the p-PKM2 (Y105), PKM2, HK I, PFKP, after co-cultured with H1299 donor cells for 24 h. The H1299 donor cells were divided into 3 group: empty vector with SiRNA control, GFP-R273H with SiRNA control and GFP-R273H with SiAP1B1. **J** Jurkat con and expressing-p53^R273H^ cells in normal Glucose media (NG, 2000 mg/L) or in low glucose media (LG, 40 mg/L) for 24 h. The statistical diagram of apoptosis was shown as mean ± s.e.m, compared to Con in LG group, according to the flow cytometer analysis of apoptosis data. **K** WB detection of apoptosis markers, cleavage-PARP and cleavage-Caspase3, in Jurkat con and expressing-p53^R273H^ cells in normal Glucose (NG, 2000 mg/L) or in low glucose (LG, 40 mg/L) for 24 h.

The function of T lymphocytes is closely associated with glucose metabolism and activated T effector lymphocytes utilize aerobic glycolysis for energy metabolism [32, 33]. To investigate whether the secreted p53^R273H^ in TME affects aerobic glycolysis in Jurkat T lymphocytes in vitro, we analyzed the extracellular acidification rate (ECAR) of Jurkat cells supplemented with the equal exosome derived from HT-29 NC or shp53 cells for 18 h and the expression of p53 R273H in exosome detected by WB was shown in Fig. S5A. The results showed that Jurkat cells with HT-29 shp53-exo group increased aerobic glycolysis, including glycolysis and glycolytic capacity compared with Jurkat cells supplemented with HT-29 shp53-exo group (Figs. 5A and S5B). Conversely, we extracted the exosome from H1299 con or R273H cells and detected the expression difference of p53^R273H^ in exosomes by WB (Fig. S5C). Subsequently, ECAR measurement showed that Jurkat with 1299 R273H-exo inhibited the glycolysis and glycolytic capacity (Figs. 5B and S5D). To further investigate the effects of p53 ^R273H^ on glycolysis of Jurkats, we constructed the stable overexpression p53^R273H^ Jurkat cells to perform ECAR measurement and the results were consistent with R273H in exosomes decreased the ability of glycolysis (Figs. 5C and S5E). Furthermore, analysis of the conditioned media revealed that Jurkat R273H cells showed lower levels of lactate production and glucose consumption, compared with the control group (Fig. 5D). Further analysis revealed that the level of phosphorylated-pyruvate kinase M2 (p-PKM2) (Tyr105) was decreased in Jurkat GFP-R273H cells (Fig. S5F). In addition, the glycolytic enzymes hexokinase-I (HK-I) and platelet isoform of phosphofructokinase (PFKP) were reduced in the Jurkat with overexpression of p53^R273H^ (Fig. S5F). Besides, we used a co-culture system that transfers secreted mutant p53 from H1299 cells to Jurkat T lymphocytes. H1299 cells were seeded in the bottom chamber and transfected with GFP or GFP-p53^R273H^ for 24 h, while Jurkat T lymphocytes were seeded in the top chamber. Co-IP assays were used to detect the endocytosis of GFP-p53^R273H^ in the recipient cells (Fig. 5E). WB analysis of the recipient cells revealed downregulation of some rate-limiting glycolytic enzymes, including p-PKM2 (Tyr105), PFKP, and HK-I (Fig. 5F). More importantly, to demonstrate the endogenous p53^R273H^ effects on the glucose metabolism, we perform the HT-29 NC or shp53 cells to co-culture Jurkat cells and the results showed that Jurkat cells in HT-29 shp53 group promotes the expression of p-PKM2 Y105, HK I and PFKP (Fig. 5G).Thereafter, to solidate the hypothesis that the secretion of mutant p53 affects the metabolism change of Jurkat, we took two strategies to inhibit the secretion of p53^R273H^ by the means of performing the p53R273H LL25AA plasmid or knocking down the AP1B1 using siRNA in tumor cells. we found that inhibition of p53^R273H^ secretion by the two ways in H1299 cells could rescue the glucose metabolism change by upregulation of p-PKM2 Y105, HK I and PFKP in Jurkat cell with the co-culture system (Fig. 5H, I). Altogether, these results indicate that tumor-derived p53^R273H^ inhibits aerobic glycolysis in T lymphocytes by reducing the expression of p-PKM2, PFKP, and HK-I. Given that p53^R273H^ had an inhibitory effect on aerobic glycolysis in Jurkat cells, we next tested the impact of metabolic stress. Low glucose (LG, 40 mg/mL) was used to culture the Jurkat T lymphocytes to mimic the metabolic stress of the tumor environment. Cells expressing mutant p53 cultured in LG demonstrated higher apoptosis rates than the control as detected by flow cytometry. By contrast, there was no significant difference in the apoptosis rate between normal and LG groups of Jurkat Con cells (Figs. 5J and S5H). Consistently, Jurkat p53^R273H^ under LG showed upregulation of apoptosis markers, such as cleavage PARP and cleavage Caspase 3 by WB analysis (Fig. 5K). These results indicate that mutant p53 promotes apoptosis of lymphocytes under glucose deficiency conditions in vitro.

### Inhibiting the secretion behavior of mutant p53 partially reverse the immunosuppressive status of CD4^+^ T lymphocytes in the tumor microenvironment

**Fig. 6:**
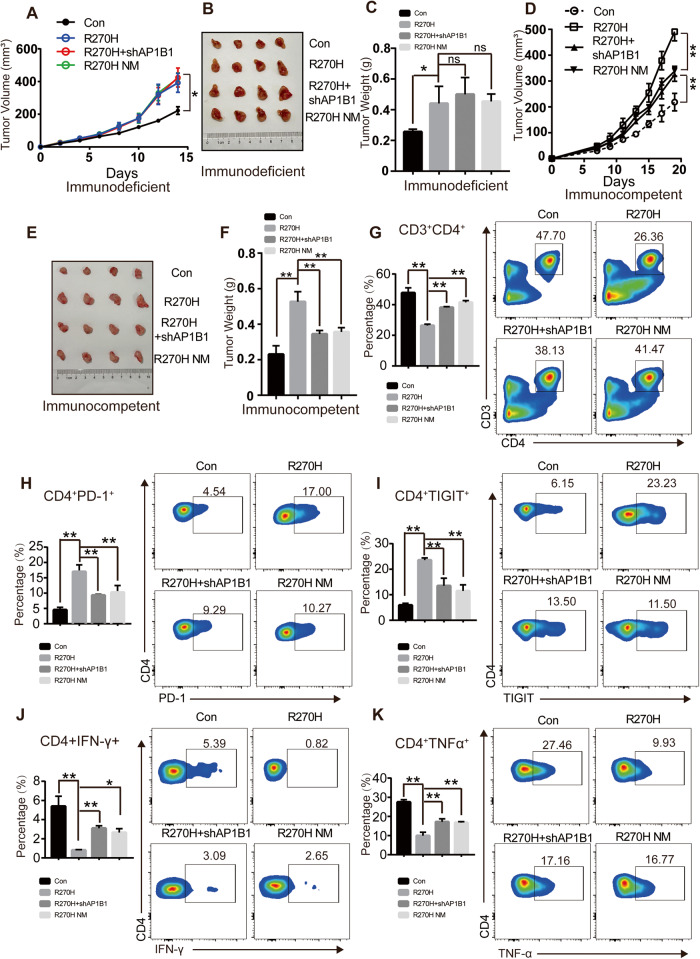
Inhibiting the secretion behavior of mutant p53 partially reverse the immunosuppressive status of CD4^+^ T lymphocytes in the tumor microenvironment. **A** 1.0 × 10^5^ 4T-1 cells expressing Con, R270H, R270H in combination with shAP1B1 (R270H+shAP1B1) or R270H NM were injected subcutaneously into female immunodeficient BALB/c null mice. Tumor volume was monitored and measured manually using slide calipers every other day (*n* = 4). **B**, **C** Representative images and weight of tumor in 4T-1 Con, R270H, R270H+shAP1B1 and R270H NM group transplanted into the immunodeficient BALB/c null mice. **D** 5.0 × 10^5^ 4T-1 cells expressing Con, R270H, R270H R270H + shAP1B1 or R270H NM were injected into the mammary gland of female immunocompetent BALB/c mice. Tumor volume was monitored and measured manually using slide calipers (*n* = 4). **E**, **F** Representative images and weight of tumor in 4T-1 Con, R270H, R270H+shAP1B1 and R270H NM group in the immunocompetent BALB/c mice. **G** Representative graphs show quantification of infiltrated CD3 + CD4 + T helper lymphocyte by flow cytometry analysis in 4T-1 Con, R270H, R270H + shAP1B1 and R270H NM tumors. **H**, **I** Flow cytometry analysis of the exhaust markers (PD-1 and TIGIT) of CD4 + T lymphocyte derived from TME in 4T-1 Con, R270H, R270H + shAP1B1 and R270H NM tumors. **J**, **K** Representative quantitation of intracellular TNF-α and IFN-γ staining in CD4 + T lymphocyte by flow cytometry in stimulation with PMA/Ionomycin for 4 h, isolated from 4T-1 Con, R270H, R270H + shAP1B1 and R270H NM tumors.

To further illuminate the effects of mutant p53 secretion behavior on tumor microenvironment to exert pro-tumor growth, we constructed 4T-1 cells with knocking-down AP1B1 by shRNA in combination with R270H or alone overexpression of R270H NM (mutated LL28AA in mice) identified by WB in Fig. S6A and the above cells were the deficient types of p53^R270H^ secretion. Then we injected the cells into subcutaneously transplanted tumor in immunodeficiency BALB/c nude mice and recorded the tumor growth rate. After 16 days, the R270H group grew much faster than Con group and showed the no differences with the inhibition p53 secretion group (Fig. 6A). Similarly, the weight of tumors is nearly no difference among R270H, R270H+shAP1B1 and R270 NM group, but the Con group was the least of all (Fig. 6B, C). Analysis of tumor samples by immunohistochemical (IHC) detection of the proliferation marker, PCNA, we found that PCNA is highly expressed in the three groups of R270H, R270H + shAP1B1 and R270 NM compared with the control group, but there were no differences among the three groups (Fig. S6B). These data indicate that inhibition of mutant p53 secretion in immunodeficiency mice have no proliferation change compared with p53^R270H^.

To solidify the hypothesis that the accelerated tumor growth in R270H tumors is partially attributed to the secretion behavior, which suppressed the CD4 + T lymphocyte-mediated anti-tumor immunity, 4T-1 Con, R270H, R270H + shAP1B1 and R270H NM cells were used to construct the orthotopic breast cancer xenograft model at the mammary pad fat in BALB/c immunocompetent mice. Mutant p53^R270H^ increased the tumor growth rate compared with Con group, whereas the R270H+shAP1B1 group or R270H NM group showed slower growth rate compared with R270H, but faster than Con group (Fig. 6D). Also, the tumor size of R270H+shAP1B1 group or R270H NM was between R270H and Con group, but there is no difference in the two secretion inhibition groups (Fig. 6E, F). Representative images of H&E staining in the tumor group were shown in the Fig. S6D. The data demonstrated that blocking of p53^R270^ secretion inhibits the tumor growth in immunocompetent mice. Furthermore, assessment of CD3 + CD4 + T lymphocyte infiltration by Flow cytometry analysis, the p53 secretion inhibition group (R270H + shAP1B1 group or R270H NM) showed a decrease recruitment than normal secretion group (R270H) and an increase than negative control group (Fig. 6G). To detect the exhausting status of CD4 + T lymphocytes by the marker, PD-1 and TIGIT, we found that unnormal secretion group, namely R270H + shAP1B1 group or R270H NM, could rescue the high expression of the markers, but still showed the much higher than control group (Fig. 6H, I). Moreover, staining for intracellular TNF-α and IFN-γ derived from CD4 + T lymphocyte under stimulating conditions that occurred in R270H tumors was decreased and this result was rescued by blocking the p53 R270H secretion (Fig. 6J, K). Overall, our data indicate that by blocking the secretion behavior, the effect of mutant p53 on pro-tumor can be partially reversed by improving the quantity and function of CD4 + T lymphocyte, thus leading to restrain the tumor growth.

## Discussion

Although several studies have demonstrated the intracellular effects of oncogenic mutant p53 in tumor growth, invasion, and metastasis as well as anoikis resistance [34–37], the crosstalk between mutant p53-driven tumor development and TME has gained increasing interest. The presence of WT or mutant p53 in the EVs greatly increases their biological function. For instance, p53 (WT or mutant) regulates exosomal secretion by participating in exosome biogenesis, promoting exosome secretion, and altering exosome content to modify cell secretome and remodel TME [38–41]. Moreover, mutant p53 is secreted into the TME by several tumor cells, including cancer-associated fibroblasts [42, 43]. A previous study demonstrated the biological role of EV-mediated p53^WT^ secretion [22]. Another study found that mutant p53 is present in etosomes and that p53 packaging into EVs is dependent on HSP90 [23]. NTA, TEM, and WB analyses, in our study, revealed the presence of p53^R273H^ in HT-29 cells, suggesting that TDE-mediated secretion of mutant p53 is an essential process for tumor progression.

Classically-secreted proteins contain an N-terminal signal peptide and are trafficked through the Golgi complex [44]. In this study, we found that the dileucine motif of p53 is required for its secretion and that mutations in the dileucine motif interfere with the interaction between p53 and β-adaptin, thus preventing its effective release. Moreover, our study demonstrated that mutant p53 packaging into the exosomes is dependent on β-adaptin in the cytosol, which is consistent with the characteristics of β-adaptin cargo [45, 46]. Therefore, these results suggest an association between the N-terminal dileucine motif of p53 and exosome secretion. Considering the complex process of exosome and EV packaging, the regulatory mechanisms of mutant p53 secretion needs to be explored further.

Previous studies have demonstrated that posttranslational modifications of p53, such as phosphorylation, acetylation and methylation, are activated in response to DNA damage and other factors to sustain cell homeostasis [47–49]. However, whether the posttranslational modification of p53 promotes its secretion remains unclear. We find that phosphorylation of Ser-20 acts as a switch to regulate the secretory behavior of p53/mutant p53. From this new perspective, phosphorylation of Ser20 mediates the reduced secretion of p53, leading to intracellular accumulation, and this may be a protective mechanism to sustain homeostasis. More importantly, phosphorylation of mutant p53 Ser20 decreases its secretion and prevents the regulation of remodeling in the tumor microenvironment that allows immune evasion. Furthermore, CHK2 phosphorylates mutant p53 Ser20 and may induce a conformation change that conceals the dileucine motif (aa 21–26). This would disrupt the interaction of mutant p53 with β-adaptin and lead to reduced secretion of mutant p53. Conversely, deficiency of CHK2 induces the secretion of mutant p53 by revealing the binding site for β-adaptin. Our results reveal a novel link between the dileucine motif and CHK2 mediated-phosphorylation of p53 Ser20 to affect its secretion process with the mechanism of change in interaction with β-adaptin.

As we all known that TME create a pro-tumor surroundings, our study also revealed that mutant p53 secretion is enhanced by chemokines or oncogenic Ras, suggesting that mutant p53 secretion is actively driven by intracellular factors and that mutant p53 acts passively within the TME. Several studies have demonstrated that, in cancer, mutant p53 and mutant p53-ID4 complex promote *VEGF* gene expression via the HIF1/VEGF-A pathway or MALAT1/VEGF-A pathway, respectively [50, 51]. Among these factors, VEGF-A could participate in a feedforward loop to facilitate the exosome-mediated secretion of mutant p53. Therefore, mutant p53 secretion may be modulated by VEGF-A in a both paracrine and autocrine manner.

Several studies have highlighted the key role of WT and mutant p53 in TME remodeling. Pharmacological activation of p53 restrains tumor immune evasion and facilitates anti-tumor immunity by engaging the cGAS/STING/IFN-γ pathway to activate innate immune response [24, 52]. Moreover, the oncogenic mutant p53 exerts an immunosuppressive environment in multiple biological processes, including induction of M2 polarization of macrophages, increase in tumor-associated neutrophil infiltration, decrease in cytotoxic CD8^+^ T cells and helper CD4^+^ T cell infiltrations, and increase in fibrosis [25, 38, 53, 54]. Consistent with the previous studies, our study demonstrated that mutant p53 disabled the CD4^+^ T cells, thus promoting their immunosuppressive function and facilitating tumor progression in vivo. We observed that secretion deficiency types of mutant p53 could partially rescue the CD4 + T lymphocytes infiltration in TME and tumor phenotypes, which may be a potential point to promote immune infiltration in TME to restrain the tumor progress. Therefore, it is possible that focusing on suppression of mutant p53 secretion by some means may improve the immune status to delay tumor progression.

The Warburg effect on glucose utilization is an important characteristic of cancers and is positively regulated by the mutant p53/p-mTOR/p-PKM2 axis [55]. In contrast with some reports, our study demonstrated that the tumor-derived mutant p53 suppresses aerobic glycolysis in lymphocytes, as seen in the H1299 cells (Fig. S5G). However, these results were consistent with some previous reports demonstrating that mutant p53 in nontumorigenic cells suppressed glycolysis [56]. These results suggest that mutant p53-mediated metabolic regulation differs between tumors and normal cells. In nontumorigenic cells, mutant p53 increases the apoptosis rate of lymphocytes under nutrition deficiency, indicating that mutant p53 can remodel the TME via metabolic changes.

The secretion of mutant p53 allows crosstalk between cancer cells and TME; however, mutant p53 is not the only cancer-derived factor that participates in TME remodeling. Emerging studies have suggested that targeting secreted-oncogenic proteins may reverse tumor immune evasion, and our study provides the theoretical basis for the development of precision cancer therapies targeting TDE-mediated p53 secretion.

## Materials and methods

### Chemicals and reagents

Dulbecco’s modified eagle medium (DMEM), Roswell Park memorial institute (RPMI) 1640 medium, penicillin/streptomycin, fetal bovine serum (FBS), and trypsin were purchased from Invitrogen. The antibodies used in this study were as follows: anti-p53 (sc-126, sc-6243) and anti-β-adaptin from Santa Cruz Biotechnology; anti-GFP from Genscript; anti-p-p53 (Ser20), anti-p-CHK2 (Thr68), anti-p-PKM2 (Tyr105), anti-PKM2, anti-PFKP, anti-HK-I, anti-cleaved-PARP, anti-cleaved-caspase 3, anti-Lamin B1, anti-p53 (#2527), anti-mTOR, and anti-p-mTOR (Ser2448) from Cell Signaling technology; anti-FLAG (clone M2), anti-β-actin, anti-α-tubulin, and anti-GAPDH from Sigma; anti-CHK2 from Millipore, and anti-p53 (10442-1-AP) and anti-AP1B1(16932-1-AP) from Proteintech. The antibodies were diluted according to the manufacturers’ instructions. Doxorubicin and bFGF were purchased from Sigma, and human VEGF-A protein was purchased from Proteintech. 10 Gy of irradiation was generated by the Rad Source R1800Q irradiator (Rad Source technologies, Buford, USA).

### Plasmid construction and viral infection

Plasmids of FLAG-p53^WT^, CHK2, K-RAS G12V, H-RAS G12V, and β1-adaptin were stored in our laboratory and confirmed by sequencing. The GFP-p53 mutants, including R175H, R248W, and R273H, were provided by Dr. Zhi-Xiong Jim Xiao (Sichuan University, Chengdu, China). The mutant p53^LL25AA^ was constructed by using a QuickChange site-directed mutagenesis kit. The mutants p53^DelN94^ and p53^DelC101^ were ligated into the BamHI/XhoI site of the pcDNA3.1 plasmid with a FLAG epitope. All the plasmids were confirmed by sequencing (GenScript, Nanjing, China).

For lentiviral infection, the control, p53^R273H^-expressing lentivirus, p53 shRNA (in human) lentivirus and AP1B1 (in mouse) shRNA lentivirus were purchased from GeneChem (Shanghai, China) and Trp53^R270H^ and Trp53^R270H LL28AA^ (NM)-expressing lentivirus was purchased from Syngentech (Beijing, China). Stable overexpression or knocking-down cell lines were selected by puromycin and the expression was confirmed by WB analysis.

### Cell culture and transfections

HEK293, and KLE cells were maintained in DMEM supplemented with 10% FBS; HCT116, H1299, 4T-1, and Jurkat cells were maintained in RPMI 1640 medium supplemented with 10% FBS; and HT-29 cells were maintained in McCoy’s 5 A medium supplemented with 10% FBS. For plasmid transfection, cells at 60% confluency were transfected with jetPRIME (Polyplus-transfection, USA), and after 24–48 h of incubation in the conditioned medium, the cells were harvested for analysis. The siRNA target sequence of β1-adaptin was as follows: 5’-CCACTCAGGACTCAGATAA-3’ (Ribobio, Guangzhou, China).

### WB analysis

The cells were lysed on ice for 30 min using IP lysis buffer (50 mM Tris-HCl, 1 mM EDTA, 1% Triton X-100, 150 mM NaCl, and 1.25% C_24_H_39_NaO_4_). After centrifugation at 14,000 rpm for 20 min, the supernatant was obtained and the protein concentration was quantified by Coomassie brilliant blue G-250 staining. Approximately 30–50 μg of protein was separated by 8% sodium dodecyl sulfate-polyacrylamide gel electrophoresis (SDS-PAGE) and transferred to polyvinylidene fluoride membranes (Millipore) for 2 h at 80 V. The membranes were blocked with 5% bovine serum albumin (BSA) in tris-buffered saline with Tween 20 (TBST) for 1 h at room temperature (RT) and incubated with primary antibodies at 4 °C overnight with slight shaking. The membranes were washed several times with TBST at RT and then incubated with HRP-conjugated second antibodies for 2 h at RT. Lastly, the protein bands were detected by using the enhanced Chemi-luminescence detection kit and visualized via DNR western blot direction system.

### Co-IP and IP analyses

For Co-IP analysis, 2 mg of protein lysate was mixed with primary antibodies and incubated at 4 °C for 3 h, followed by incubation with A/G-sepharose beads (Santa Cruz) at 4 °C overnight. The complex was harvested by centrifugation at 700 g for 5 min at 4 °C and washed 3 times with cold phosphate-buffered saline (PBS). Thereafter the complex was boiled in a 2× loading buffer for 10 min and the supernatant was subjected to SDS-PAGE.

To verify the secretion of WT and mutant p53 in the culture media, the media was centrifuged at 1000 rpm for 5 min and the supernatant was filtrated through a 0.22 μm membrane filter. The filtrate was then sequentially subjected to IP and WB assays.

### Exosome isolation

HT-29 and H1299 cells were grown in their respective media supplemented with 10% exosome-free FBS, in which the exosomes were removed from the FBS by ultracentrifugation. The culture medium was centrifuged at 2000 g for 20 min and filtered through a 0.22 μm filter to remove cell debris. Thereafter, the exosomes were extracted from the filtered media using the Total Exosome Isolation Reagent (Cat. 4478359, Thermo Fisher Scientific), according to the manufacturer’s instructions. The harvested pellet was then lysed using IP lysis buffer, and the protein quantity in the exosomes was measured using a bicinchoninic acid assay (BCA) kit (Dingguo, Guangzhou, China).

Ultracentrifugation was conducted as follows: the filtered medium was first centrifuged at 100,000 g for 80 min at 4 °C in an ultracentrifuge. Thereafter, the pellet was resuspended in PBS and centrifuged again for 80 min at 4 °C. After centrifugation, the pellet was dissolved in cold PBS and the protein concentration was quantity using the BCA kit. The exosome samples were stored at −80 °C. CD63, CD9, CD81, Alix, and TSG101 (Proteintech, Wuhan, China) were considered as the positive markers, while Calnexin (sigma) was considered as a negative marker for exosomes for the WB analysis. Furthermore, the isolated exosomes were subjected to NTA analysis and observed under TEM (APTBIO, Shanghai, China).

### Immunofluorescence assay

HEK293 cells were first transfected with FLAG-p53^WT^ and FLAG-p53^R273H^, then fixed using 4% paraformaldehyde and permeabilized by PBS containing 0.1% Triton X-100. The cells were first incubated with anti-FLAG and anti-β-adaptin antibodies (1:100) and subsequently incubated with secondary fluorescence-labeled antibodies (1:250; Invitrogen) and DAPI (Sigma, 1:1000). The fluorescence was visualized by the Nikon C2 plus confocal microscope.

### Lactate production and glucose consumption assays

The cells (2 × 10^5^) were seeded in 6-well plates and cultured in RPMI 1640 medium overnight. Thereafter, the culture medium was replaced with serum-free RPMI 1640 medium (without phenol red) with 5% BSA and incubated for 8 h. This culture medium was used to determine lactate concentration using the Lactate Assay Kit (Nanjing Jiancheng Bioengineering, Nanjing, China), according to the manufacturer’s instructions. The rate of lactate production was measured and normalized to that of the control group. For the glucose consumption assay, the cells were cultured in FBS-free RPMI 1640 medium with 0.2% BSA for 12 h, and the glucose concentration in the medium was measured using the Glucose Assay Kit (Rongsheng Bio-tech, Shanghai, China). Glucose consumption was determined by calculating the difference between the initial and the residual glucose concentration in the culture medium. The glucose consumption was normalized to the protein concentration and compared with that of the control group.

### ECAR measurement

ECAR was performed using the seahorse XF24 Analyzer. The cells were seeded in the plate and cultured in the media treatment with equal exosome containing p53^R273H^ or control at 37 °C and 5% CO_2_ for 18 h. Thereafter, the culture medium was replaced with FBS- and sodium bicarbonate-free assay medium, and the plates were incubated at 37 °C without CO_2_ for 1 h. To conduct ECAR measurement, the additions were treated with the cells at the concentrations of 11 mM glucose, 1 μM oligomycin, and 20 mM 2-DG, respectively.

### In vivo tumor implantation

4T-1 control or Trp53^R172H^ or Trp53^R270H^-expressing cells were digested by trypsin and washed by PBS buffer. Then 5.0 × 10^5^ cells per group in 100 μl PBS were injected into the mammary fat pad of female BALB/c mice (8-weeks-old) after anesthesia. The mice were monitored and the tumors were measured once every 2 d using Vernier calipers.

4T-1 control, 4T-1 R270H (infection with control or shAP1B1) and 4T-1 R270H NM were trypsinized and washed with PBS. After anaesthetizing mice, fix mice, disinfected and cut the skin, and inject the 5.0 × 10^5^ of the above cells into the mammary fat pad of female BALB/c mice. Subsequently, the skin was sutured and the mice were monitored and the tumors were measured once every 2 d using Vernier calipers.

4T-1 control, 4T-1 R270H (infection with control or shAP1B1) and 4T-1 R270H NM were trypsinized and washed with PBS. 1.0 × 10^5^ of the above cells were injected into the subcutaneous BALB/c nude mice to establish the transplanted tumor model. the mice were monitored and the tumors were measured once every 2 d using Vernier calipers. The tumor volume was calculated (length × width^2^/π). All animal experiments were approved by the Institutional Animal Care and Use Committee of China Medical University.

### Immunohistochemistry and H&E staining

For immunohistochemistry assays, the samples from the animal experiments were fixed in the 4% paraformaldehyde and prepared the paraffin slices according to the procedures. The samples were dewaxing, hydrating and performed high pressure antigen retrieval with citrate buffer (pH 6.0). To rule out the endogenous peroxidase, 3% hydrogen peroxide were used for 30 min and then 10% goat serum were used to blocking the nonspecific staining for 1 h. The sections were incubated with primary-anti-PCNA (CST, 2586 S,1:1000) overnight at 4 °C. Subsequently, the slides were stained with biotinylated secondary antibody and streptavidin-biotin peroxidase and DAB chromogenic reagent according to the ultrasensitive SPIHC Kit (Maixin Inc., China). The sections were stained with hematoxylin and sealing for further data analysis.

For H&E staining, the sections were deparaffinzing in xylene and hydrating in graded ethanol. Then the slides were stained with hematoxylin and eosin respectively according to the procedure.

### Flow cytometry

To assess apoptosis, the cells were cultured for 24 h in RIPM 1640 medium containing normal glucose (2 g/L) or low glucose (40 mg/L) levels. The cells were centrifuged and resuspended in 0.5 ml annexin V-binding buffer (KeyGEN Biotech, China). Thereafter, 5 μl annexin V-APC and 7-AAD were added to the samples and incubated at RT for 10 min in the dark. The samples were then analyzed on a FACS Caliber flow cytometer (BD Biosciences, US).

The lymphocytes from 4T-1-injected BALB/c mice were isolated as follows: the tumor tissues from the mice were sectioned and digested with 2 mg/ml collagenase IV and 100 ng/ml DNase I (sigma) at 37 °C for 30 min. The tissues were then added to RPMI 1640 media supplemented with 10% FBS and 0.5 mM EDTA and separated by discontinuous 30–70% Percoll (GE Healthcare). After stimulation with PMA/Ionomycin and BFA (sigma) for 6 h at 37 °C, the cells were harvested for surface staining and intracellular staining (BD Pharmingen), according to the manufacturer’s instructions. The antibodies were anti-CD45-BV510, anti-CD45-APCcy7, anti-CD3-APC, anti-CD3-BV650, anti-CD4-FITC, anti-CD4-PEcy7, anti-PD-1-BV785, anti-PD-1-PE, anti-TIGIT-BV421, anti-TCR α/β-PE anti-IFN-γ-BV785, anti-IFN-γ-APC, anti-TNF-α-BV421 and anti-TNF-α-APC (BioLegend). All the data were collected by BD FACS Celesta flow cytometry and processed by Flow Jo software.

### Statistical analysis

Data are presented as mean ± SD/SEM and analyzed by GraphPad Prism software. The significance of differences was analyzed by one-way ANOVA analysis of variance and independent samples *t* test. *P*-value < 0.05 was considered statistically significant.

### Supplementary information

supplementary Figures

supplemtary figure legend

## Data Availability

All data generated or analyzed during this study are included in this article and its Supplementary Information Files.
