# Supplementary figures and images for: Regulated secretion of mutant p53 negatively affects T lymphocytes in the tumor microenvironment
Source: Oncogene. 2023 Nov 11;43(2):92–105. doi: 10.1038/s41388-023-02886-1 (PMC10774126; doi:10.1038/s41388-023-02886-1)

Supplementary Figures


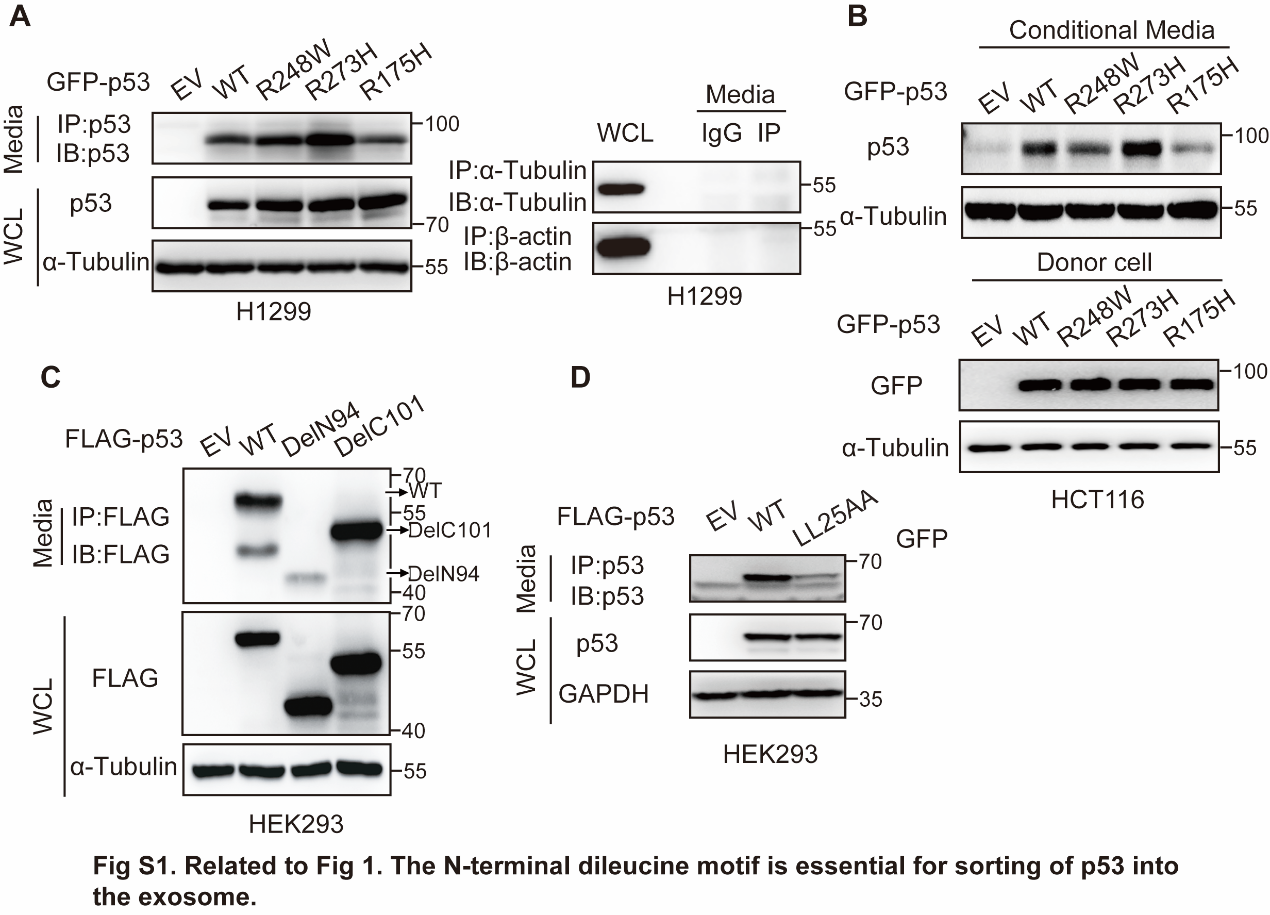


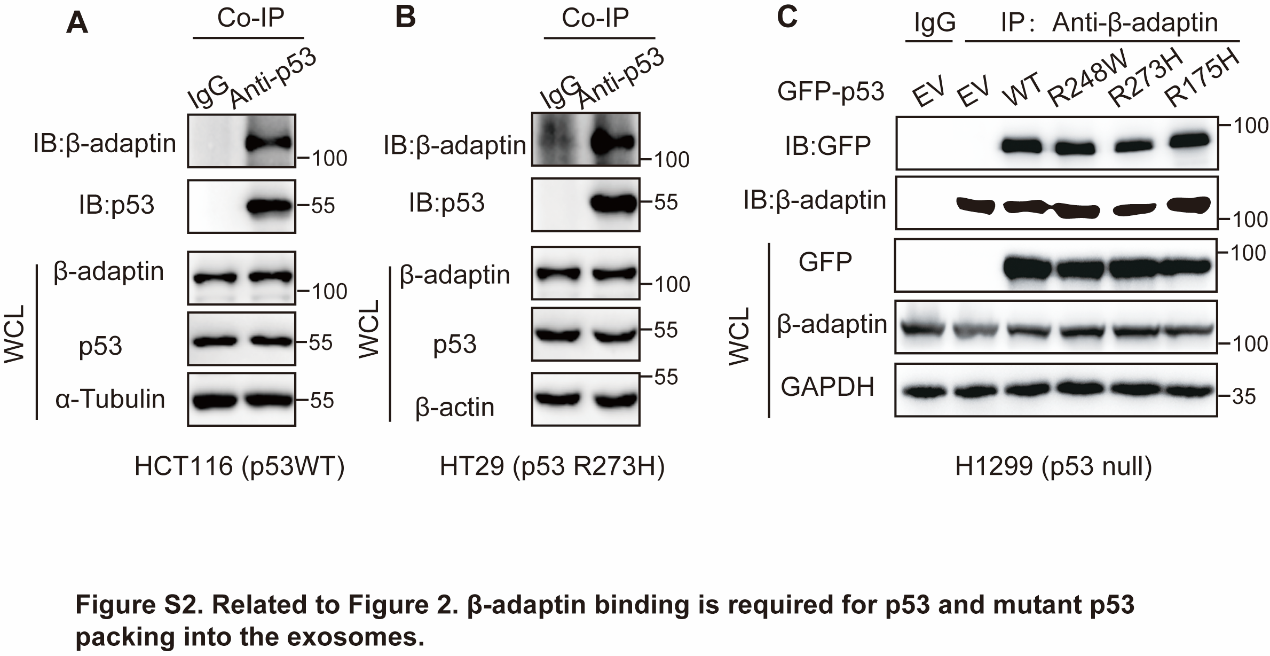


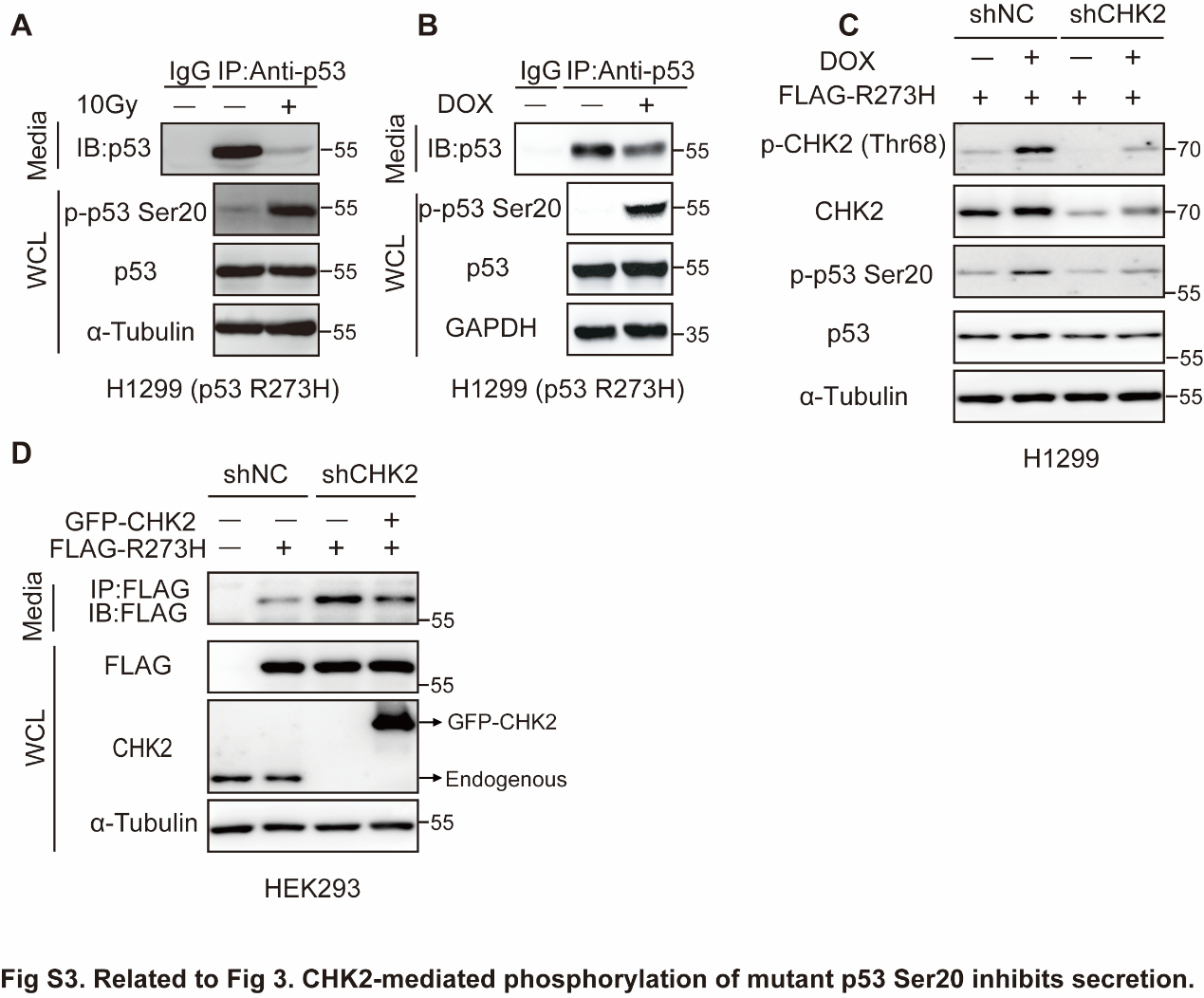


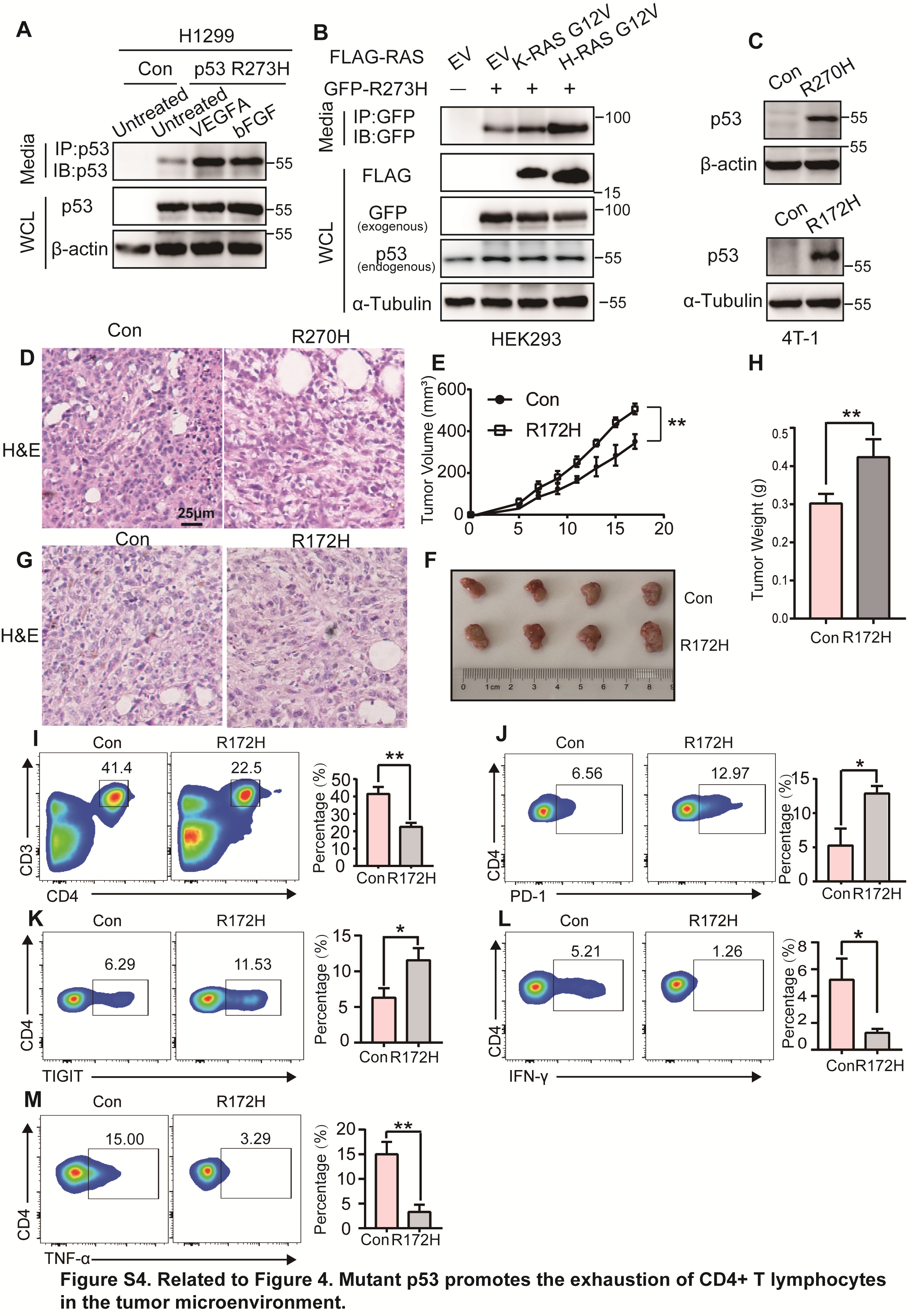


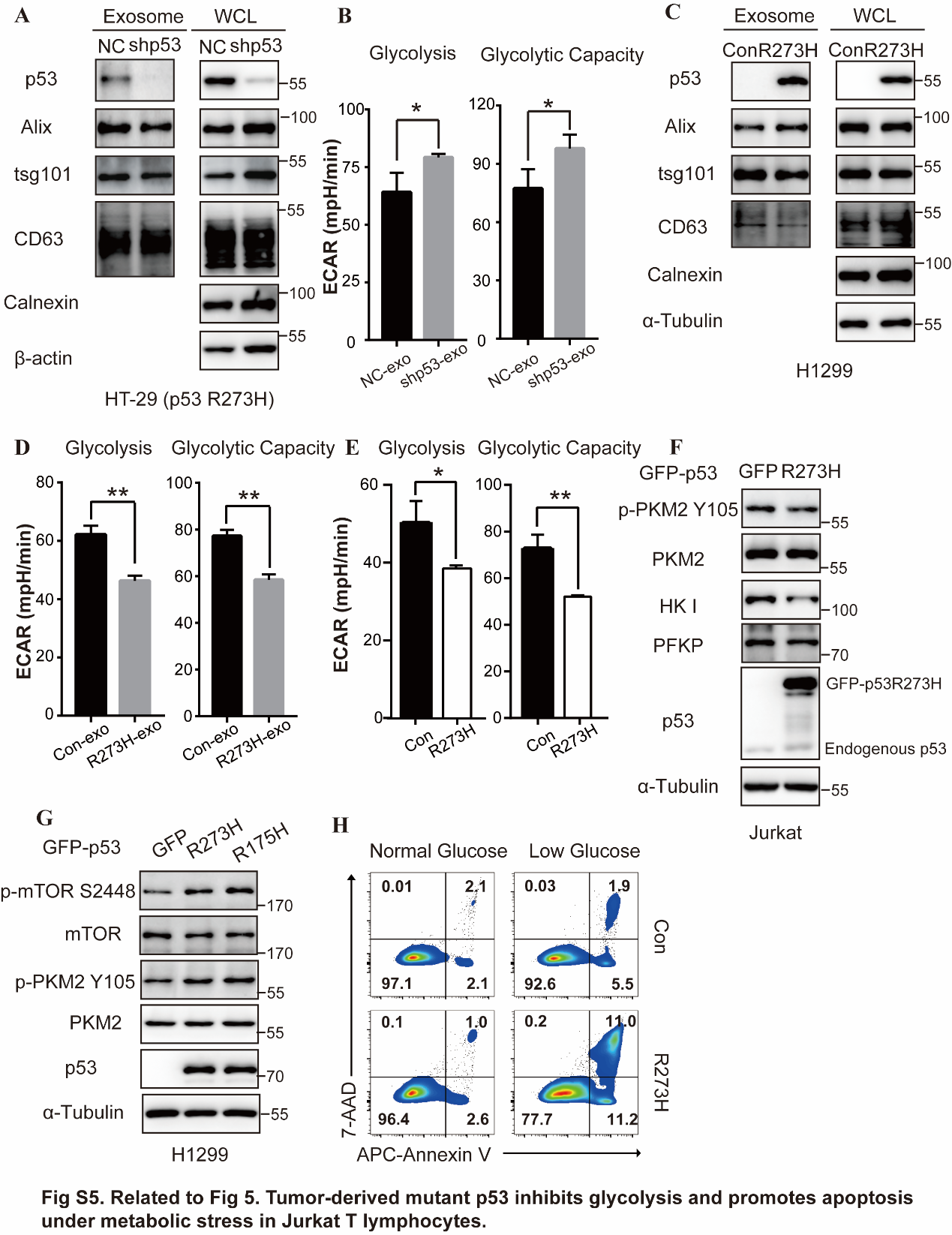


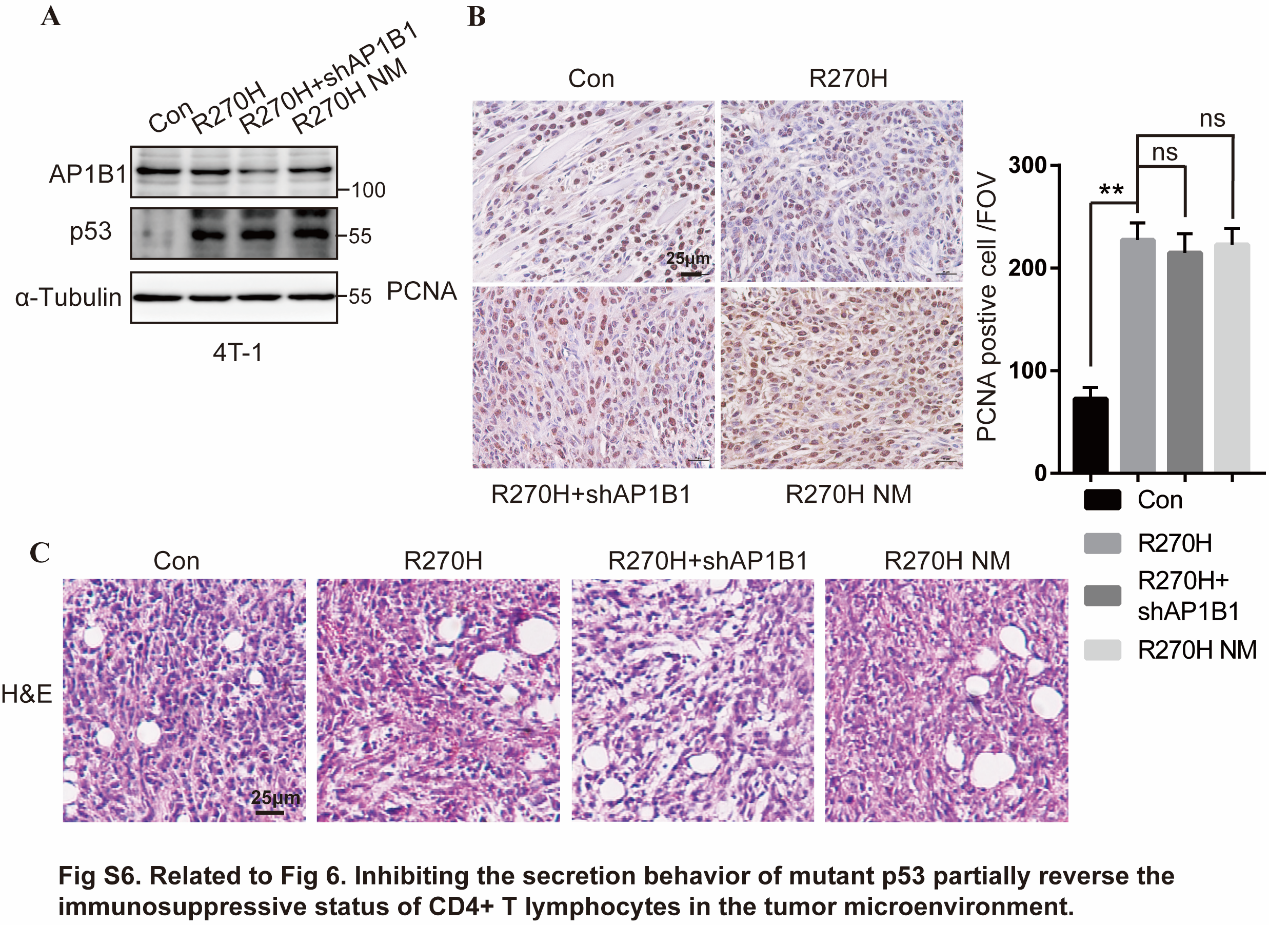

Supplement: Supplementary file 1 — supplementary Figures [file 41388_2023_2886_MOESM1_ESM.doc]
