# Supplementary material for: Regulated secretion of mutant p53 negatively affects T lymphocytes in the tumor microenvironment
Source: Oncogene. 2023 Nov 11;43(2):92–105. doi: 10.1038/s41388-023-02886-1 (PMC10774126; doi:10.1038/s41388-023-02886-1)
Supplement: Supplementary file 2 — supplemtary figure legend [file 41388_2023_2886_MOESM2_ESM.doc]

**Fig S1. Related to Fig 1. The N-terminal dileucine motif is essential for sorting of p53 into the exosome.**

**(A)** Immunoprecipitation (IP) assay of cell culture supernatants of H1299-expressing empty vector, p53^WT^, p53^R248W^, p53^R175H^ and p53^R273H^ cells using anti-p53 antibodies. α-tubulin and β-actin were used as the negative controls. Western blot (WB) analysis of the whole cell lysates and media fractions. **(B)** The conditioned media (CM) was a mixture of fresh culture media from tumor cells transfected with or without exogenous WT or mutant GFP-p53 as the donor cells. HCT116 cells were cultured in the CM for 2 h and subjected to WB analysis. **(C)** The indicated plasmids were overexpressed in HEK293 cells. IP analysis of FLAG-p53^WT^，p53^DelN94^ and p53^DelC101^ in the HEK293 cell culture supernatant, using anti-FLAG antibodies, followed by WB analysis. **(D)** IP analysis of FLAG-p53^WT^ and p53^LL25AA^ in the HEK293 cell culture supernatant, by using anti-p53 antibodies.

**Fig S2. Related to Fig 2. β-adaptin binding is required for p53 packing into the exosome.**

**(A, B)** Immunoprecipitation analysis of the endogenous p53 and β-adaptin in p53^WT^-expressing HCT116 **(A)** and endogenous p53^R273H^-expressing HT-29 **(B)**, using anti-p53 antibodies. **(C)** Co-immunoprecipitation analysis of exogenous wildtype or mutant p53 (p53^R248W^, p53^R273H^, and p53^R175H^) from H1299 cells, using anti-β-adaptin antibodies, followed by western blot analysis.

**Fig S3. Related to Fig 3. CHK2-mediated phosphorylation of mutant p53 Ser20 inhibits secretion.**

(**A**, **B**) Expression of p53^R273H^ immunoprecipitated by anti-p53 antibodies from the H1299 stable over-expression of p53^R273H^ cell culture supernatant after treatment without or with 10Gy IR for 4h (**A**) and 500nM DOX for 12h (**B**). **(C)** Expression of p-CHK (Thr68), CHK2, p-p53 (Ser20) and p53 in H1299 (p53 null) knock down of CHK2 or not cells after transfection with FLAG-p53^R273H^ and subsequent treatment with or without 500nM DOX for 12h. **(D)** Analysis of exogenous FLAG-p53^R273H^ in media fraction secreted by H1299 NC, H1299 NC+FLAG-R273H, H1299 shCHK2+ FLAG-R273H and H1299 shCHK2+ FLAG-R273H+ReGFP-CHK2 group.

**Fig S4. Related to Fig 4. Mutant p53 promotes the immunosuppressive status of CD4^+^ T lymphocytes in the tumor microenvironment.**

**(A)** Stable p53^R273H^ -expressing and negative control-expressing H1299 cells were subjected to serum starvation for 4 h and then treated with 10 ng/ml VEGF-A or 50 ng/ml bFGF for 12 h. The cell culture supernatant was analyzed by immunoprecipitation (IP) to detect p53^R273H^ secretion and the lysates were analyzed by WB. **(B)** HEK293 cells were co-transfected with GFP-p53^R273H^ and FLAG-K-RAS G12V or H-RAS G12V for 36 h and then subjected to IP, using anti-GFP antibodies, followed by WB analysis. **(C）**WB shows the p53 expression in 4T-1 infected with Con or Trp53^R270H^ or Trp53^R172H^ lentivirus after selected with puromycin. **(D&G)** Representative images for H&E staining to detect the morphological structure in 4T-1 Trp53^R270H^ or Trp53^R172H^ or their respective control tumors for orthotopic transplantation model injected into the mammary fat pad of BALB/c mice (scar bar: 25μm). **(E)** Graphical quantification represents tumor growth rate in mice with time (n = 4) after an equal amount of 4T-1 control, Trp53^R172H^‑expressing cells were injected into the mammary gland of female BALB/c mice. **(F)** 4T-1 tumor-harboring mice were sacrificed on day 19. Representative images show tumor volume difference between Con and R172H. **(H)** Graphical quantification of the difference in tumor weight between Control and R172H mice (n = 4). **(I)** Flow cytometry analysis of CD45, CD3, and CD4 in T lymphocytes and the percentage of CD3^+^ CD4^+^ T lymphocytes isolated from R172H and its control mice. **(J)** Flow cytometry analysis of CD45, CD4, and PD-1 in T lymphocytes and the percentage of CD4^+^ PD1^+^ T lymphocytes isolated from R172H and its control mice. **(K)** Flow cytometry analysis of CD45, CD4, and TIGIT in T lymphocytes and the percentage of CD4^+^ TIGIT1^+^ T lymphocytes isolated from R172H and its control mice. **(L, M)** Quantification of Interferon-γ^+^ **(L)** and tumor necrosis factor-α^+^ **(M)** cells as a percentage of CD4^+^ T cells, after 4 h of stimulation with PMA/Ionomycin, isolated from R172H and control mice.

.

**Fig S5. Related to Fig 5. Tumor-derived mutant p53 inhibits glycolysis and promotes apoptosis under metabolic stress in Jurkat T lymphocytes.**

**(A)** Western blot analysis of endogenous p53^R273H^ in the exosomes isolated from HT-29 NC and shp53 cells, with Alix, tsg101 and CD63 considered positive markers and calnexin considered a negative marker of the exosomes. **(B)** The glycolysis and glycolytic capacity of Jur NC-exo and shp53-exo were calculated according to the dynamic monitoring of extracellular acidification rate. **(C)** Western blot analysis of p53^R273H^ in the exosomes isolated from stable p53^R273H^-expressing and negative control-expressing H1299 cells, with Alix, tsg101 and CD63 considered positive markers and calnexin considered a negative marker of the exosomes. **(D)** The glycolysis and glycolytic capacity of Jur Con-exo and R273H-exo were calculated according to the dynamic monitoring of extracellular acidification rate. **(E)** The glycolysis and glycolytic capacity were shown in Jur Con and R273H cells. **(F)** WB analysis of p-PKM2 (Y105), PKM2, HK-I, PFKP, p53, and α-Tubulin in Jurkat cells transient transfection with GFP and GFP-R273H. **(G)** WB analysis of p-mTOR (S2448), mTOR, p-PKM2 (Y105), PKM2, p53 and α-Tubulin in H1299 transfected with GFP, GFP-p53^R273H^or GFP-p53^R175H^. **(H)** Representative flow cytometer analysis of apoptosis data was shown in Jurkat Con and expressing-p53^R273H^ cells in normal Glucose media (NG, 2000mg/L) or in low glucose media.

**Fig S6. Related to Fig 6. Inhibiting the secretion behavior of Mutant p53 partially reverse the immunosuppressive status of CD4^+^ T lymphocytes in the tumor microenvironment.**

**(A)** WB shows 4T-1 cells infected with Con, R270H, R270H in combination with AP1B1 shRNA and R270H NM (LL28AA in mice) lentivirus after selected with puromycin. **(B)** Representative images and quantitation of tumor paraffin section stained with PCNA to detect the cell proliferation in 4T1 Con, R270H, R270H+shAP1B1 and R270H NM group injected subcutaneously into the immunodeficient BALB/c null mice (scar bar: 25μm). **(C)** Representative images for H&E staining to detect the morphological structure in 4T1 Trp53^R270H^ Con, R270H, R270H+shAP1B1 and R270H NM group for Orthotopic transplantation model injected into the mammary fat pad of BALB/c mice (scar bar: 25μm).
